# Investigating the Role and Response of Root Groups of *Hordeum vulgare* subsp. *spontaneum* Germplasm to Drought Stress

**DOI:** 10.1002/pei3.70103

**Published:** 2025-12-10

**Authors:** Hooman Shirvani, Ali Ashraf Mehrabi, Mohsen Farshadfar, Hooshmand Safari, Ali Arminian, Foad Fatehi

**Affiliations:** ^1^ Department of Agronomy and Plant Breeding, Faculty of Agriculture Ilam University Iran; ^2^ Faculty of Agricultural Sciences Shahed University Tehran Iran; ^3^ Forests and Rangelands Research Department Kermanshah Agricultural and Natural Resources Research and Education Center, Agricultural Research, Education and Extension Organization (AREEO) Kermanshah Iran; ^4^ Department of Agriculture Payame Noor University Tehran Iran

**Keywords:** root density, root structure, water stress, wild barley

## Abstract

*Hordeum vulgare* subsp. *spontaneum*, the wild progenitor of cultivated barley, shares the same chromosome number with domesticated forms and exhibits no significant biological barriers to interspecies crossing. Roots are essential for water uptake, nutrient acquisition, and structural support, making them key determinants of plant performance under drought stress. The present study aimed to investigate the diversity in root group responses to water deficit across 114 genotypes of wild barley. The experiment was conducted in an Augmented Block Design with two soil moisture regimes: normal conditions at 90%–95% field capacity (FC) and water stress at 50%–55% FC. Genotypes were classified into nine groups based on mean root length and root tissue density, calculated within a 95% confidence interval, under both moisture regimes. Discriminant analysis revealed that the three main discriminant functions explained 95.3% and 94.2% of the total variance under normal and water stress conditions, respectively. Analysis of variance revealed that the genotype × stress interaction effect was not significant for root diameter under drought stress. However, seedling length, root dry weight, root surface area density, and chlorophyll content were significant at *p* < 0.05, while all other measured traits were significant at *p* < 0.01. Mean root trait analysis demonstrated considerable variation among genotypes, indicating broad genetic diversity in root and shoot characteristics. Cluster analysis classified the root groups into three clusters under both water stress and normal conditions. These findings provide insights into the adaptive potential of wild barley roots under drought, supporting their use in breeding for stress tolerance.

## 1 Introduction

Barley ( *Hordeum vulgare* ) is a major cereal crop with rich genetic diversity and various types, such as two‐row and six‐row barley, covered and naked varieties, and differing starch compositions (Finocchiaro et al. 2023; Hagenblad et al. 2024). It is commonly used for animal feed, malting for brewing and distilling, and human consumption due to its high beta‐glucan content, which offers health benefits such as the reduction of cholesterol and blood sugar levels (Geng et al. 2022; Lukinac and Jukić 2022; Sadak et al. 2024).

Wild barley, scientifically known as *H. spontaneum* , is considered the progenitor of cultivated barley ( *H. vulgare* ), with remains of barley grains found in the Fertile Crescent suggesting domestication around 10,000 years ago (Badr et al. 2000; Tyagi et al. 2014). This close relationship highlights the importance of wild barley as a genetic resource for understanding and improving stress tolerance in cultivated barley. Wild barley and its wild relatives, including *H. spontaneum* , have shown adaptations to various environments, displaying genetic diversities that contribute to drought and salt tolerances (Nevo and Chen 2010). These adaptations make wild barley a valuable model for exploring the genetic and physiological mechanisms underlying stress resistance, particularly under water stress conditions. Variations in responses to osmotic stress have been observed between wild and cultivated barley, underscoring the importance of understanding gene expression and physiological adaptations in wild barley (Kreszies et al. 2019). Investigating these differences can provide insights into the mechanisms that could be targeted to enhance drought tolerance in breeding programs.

Wild barley has been investigated for its potential in breeding for traits such as drought tolerance, utilizing its extensive genetic resources (Barati, Majidi, Mirlohi, et al. 2018; Barati, Majidi, Mostafavi, et al. 2018). Its genetic diversity offers promising opportunities for identifying traits that could improve cultivated barley's resilience to environmental stresses. Moreover, wild barley germplasm has been employed in research focusing on traits such as root morphology, malting quality, and resistance to diseases such as leaf rust and aphids (Åhman and Bengtsson 2019; Chen et al. 2021; Grossman and Rice 2012; Ninkovic and Åhman 2009). These traits are particularly relevant to the current study, which aims to investigate root‐related traits under water stress conditions. Studies on the genetic diversity and population structure of wild barley have provided insights into its evolutionary history and ecological niche modeling (Orabi et al. 2009; Russell et al. 2014). This knowledge lays the foundation for understanding the ecological and genetic factors that influence the adaptability of wild barley.

Roots play a crucial role in the plant lifecycle and ecosystem functioning. They are responsible for various functions such as water and nutrient uptake, structural support, and adaptation to environmental stresses. The root system exhibits phenotypic plasticity (Xu et al. 2023), which allows plants to adjust their morphology in response to changing conditions such as water availability, soil nutrients, and interactions with other organisms. Fine roots, the distal parts of the root system, contribute to soil organic matter accrual and influence mineral weathering and soil microbial dynamics (El Amrani 2023). Root system architecture (RSA) plays a crucial role in plant response to water stress and normal conditions (Abdirad et al. 2022). Under water stress, deep and branched root systems aid in water and nutrient acquisition, with lateral root expansion as a common strategy (Li et al. 2017). Understanding the dynamics of RSA under stress conditions is essential for identifying traits that enhance drought tolerance, directly aligning with the objectives of this study.

Barley roots exhibit variability in morphological traits, impacting their adaptation to stress environments and overall plant development. Studies have highlighted differences between seminal and nodal roots, with nodal roots showing larger diameter, metaxylem area, and enhanced nutrient uptake capacities compared to seminal roots (Dutta et al. 2023). Root traits such as total length, diameter, and root‐to‐shoot ratio are crucial for breeding programs to develop stress‐tolerant cultivars (Wang et al. 2021). Additionally, root‐related traits such as dry weight, area, volume, and length play a vital role in determining grain yield and drought tolerance in barley, with a vigorous root system contributing to yield stability under stress conditions (Liu et al. 2020). Furthermore, root exudates and rhizosheaths are essential for soil binding and rhizosheath formation, with root hairs facilitating the release of soil‐binding polysaccharides (Barati et al. 2020). These findings underscore the importance of evaluating root morphological traits in wild barley genotypes to identify potential candidates for breeding drought‐tolerant cultivars.

Wild barley ( *H. spontaneum* ) germplasm exhibits variations in root traits under water stress and normal conditions. Studies have shown that wild barley genotypes possess extensive root systems with high root dry weight, area, volume, and length, especially under drought stress, indicating adaptability to water stress environments (Yakovleva 2023). Furthermore, wild barley genotypes have shown increased lateral root growth, deeper rooting depth, and enhanced water uptake efficiency under reduced water availability conditions, highlighting their potential for improved water stress tolerance compared to cultivated barley varieties (Khodaeiaminjan et al. 2023). Wild barley genotypes have been found to have more drought tolerance than cultivated ones, with certain genotypes from Iran showing tolerance at both vegetative and reproductive stages, making them potential candidates for breeding programs. Additionally, wild barley genotypes with high root dry weight, area, volume, and length, as well as a high root‐to‐shoot ratio, were found to have more yield stability under drought stress conditions (Barati et al. 2020). These findings provide a foundation for this study, which aims to evaluate root traits in wild barley genotypes to identify those with the greatest potential for breeding drought‐tolerant cultivars.

The root trait of wild barley ( *H. spontaneum* ) under water stress has been studied across various developmental stages. Drought stress has been shown to reduce germination percentage, primary root number, and shoot and root length of seedlings. At the vegetative stage, drought stress decreased shoot dry weight (SDW) and root dry weight (RDW), but the ratio of RDW to SDW (RSR) increased, indicating a relative enhancement of root growth compared to shoot growth under water stress conditions. At the reproductive stage, while SDW, plant height, and number of fertile tillers decreased, root length, root volume, RDW, and RSR increased as drought intensity rose (Barati et al. 2015).

Given the significance of barley production as a crucial economic crop and the growing challenges posed by climate change, including the sharp decline in rainfall and the resulting water scarcity, it is essential to conduct fundamental studies on the root trait diversity in barley. Variability in root trait among barley genotypes plays a key role in selecting varieties with optimal root traits for effective stress adaptation and resource acquisition. This research aims to investigate the role and response of root groups in wild barley ( *H. spontaneum* ) germplasm under water stress, focusing on the physiological and morphological changes in the root system. By understanding the mechanisms by which water stress influences root development, this study offers valuable insights into enhancing the resilience and productivity of barley under water stress conditions. Furthermore, through breeding programs and genetic engineering using parental species, high‐yielding and drought‐tolerant barley cultivars with more efficient root systems can be developed.

## 2 Materials and Methods

This research involved 114 genotypes of wild barley ( *H. spontaneum* ), which were collected from four western provinces of Iran (Zagros basin). The collection included 29 genotypes from Kermanshah, 28 from Kurdistan, 28 from Ilam, and 29 from Lorestan. The wild barley genotypes were collected from the western provinces of Iran, Kermanshah, Kurdistan, Ilam, and Lorestan due to their diverse climatic conditions, ranging from semi‐arid to temperate, and varying soil properties. These regions feature a combination of low fertility and deep soils, which contribute to the adaptation of wild barley to environmental stresses, such as drought. The variation in climate and soil types makes these areas ideal for studying genetic diversity, root traits, and drought tolerance mechanisms in wild barley, which are essential for developing drought tolerance barley cultivars. The gene bank code (IUGB), genotype numbers, locations/regions, and geographic coordinates are provided in the Table S1.

The experiment was carried out in the greenhouse of the Agricultural Research Center and Natural Resources of Kermanshah province under water stress and normal conditions in 2021. An Augmented Block Design with a randomized block structure was used for the experiment. The design included five replications for nine control genotypes: {genotype no. 1 (Kermanshah‐Mahidasht), 24 (Kermanshah‐Sarpol‐e Zahab), 35 (Kermanshah‐Harsin), 10 (Ilam‐Ilam), 51 (Ilam‐Mehran), 74 (Kurdistan‐Kamyaran), 34 (Kurdistan‐Bijar), 104 (Lorestan‐Kohdasht), and 113 (Lorestan‐Aligudarz)}. The design included five blocks, each containing 30 tubes, with 21 genotypes and 9 control genotypes replicated across the blocks, resulting in a total of 150 tubes per treatment and 300 tube s in the experiment. For each tube, seven seeds were initially sown. After germination, seedlings were thinned to five per tube. The experimental setup for both the water stress and normal conditions was identical in the greenhouse, ensuring that only the water stress treatment differentiated the two conditions.

Control genotypes were randomly selected to ensure adequate representation of diverse geographic regions. For each region, a minimum of two genotypes was included and designated as controls within the experimental design. This strategy provided a comprehensive basis for comparing the responses of different genotypes to water stress, and the results of these control genotypes are presented and discussed throughout the manuscript. To reduce experimental error and enhance the accuracy of comparisons, the data obtained from the experimental genotypes were corrected using the control genotypes. This correction was performed to minimize the effects of environmental factors and other uncontrollable variables.

After correcting the treatments, the genotypes were divided into nine groups based on the root length and root tissue density traits. To group the genotypes, those with root length values below the 95% confidence interval of the mean were classified as superficial genotypes, while genotypes with root lengths within the mean range were classified as semi‐deep. Genotypes with root lengths exceeding the upper limit of the mean confidence interval were classified as deep genotypes. A similar procedure was applied to the root tissue density trait, with genotypes categorized into three groups: non‐dense, semi‐dense, and dense, based on their root tissue density.

### 2.1 Investigating Root Trait in the Seedling Growth Stage

We used river sand (natural sand) for the cultivation of the genotypes. Specifically, the sand was collected from the Qarasu River in Kermanshah Province, Iran, and was carefully selected to be free from contaminants that could affect the growth of the barley genotypes. Seven seeds were planted in each tube, and after germination, the number of seedlings was reduced to five. Seedlings were provided with all necessary nutrients through a nutrient solution prepared according to standard protocols for barley cultivation. The solution contained essential macronutrients, including Nitrogen (N), Phosphorus (P), Potassium (K), Calcium (Ca), Magnesium (Mg), and Sulfur (S), along with micronutrients such as Iron (Fe), Manganese (Mn), Zinc (Zn), Copper (Cu), Boron (B), Molybdenum (Mo), and Chlorine (Cl). Nutrient concentrations were adjusted to optimal levels based on recommendations from previous studies. The pH of the solution was maintained within the range of 5.5–6.5 to ensure proper nutrient availability. The nutrient solution was renewed weekly to provide fresh nutrients throughout the experimental period.

The tubes containing the seedlings were placed in a greenhouse with natural light, where the temperature was controlled to range between 25°C during the day and 15°C at night. After six weeks, root and shoot traits were measured. For root trait measurements, destructive sampling was performed to remove the roots from the culture tube beds. The sand inside the tubes was gently washed using water pressure on a sloped surface, ensuring that all sand residues were completely separated from the roots. The aerial part, including the stem and leaves, and the roots were then separated. The culture tubes were kept in the greenhouse under natural light, with strict temperature control to maintain a daytime temperature of 25°C and a nighttime temperature of 15°C. To support optimal seedling growth, relative humidity was maintained within the standard range for controlled greenhouse environments. Additionally, light intensity was continuously monitored using a quantum sensor (LI‐COR Biosciences) to ensure it remained within the appropriate range for healthy seedling development.

### 2.2 Water Stress

To induce water stress, the field capacity (FC) of the sand and the soil moisture retention duration were first determined. Water stress was applied at two field capacity (FC) levels: normal (FC = 90%–95%) and water stress (FC = 50%–55%), based on the field capacity method. After the initial growth and establishment of the plants, water stress was applied when the plants reached the 14‐day stage (seedlings) to simulate early stress conditions. Root traits were evaluated during the early vegetative growth phase, which in barley typically spans 4–6 weeks and is critical for root development and overall plant growth (Francia et al. 2013). The experiment was conducted until the plants reached the 6‐week stage, which was the final assessment point. The stress duration lasted for four weeks. To measure field capacity, a Sartorius balance with 0.001 g precision was used to determine the weight difference in soil samples before and after drying. The field capacity was calculated by comparing the weight of the soil samples before and after drying.

### 2.3 Root Traits

Seedling fresh and dry weights, as well as root fresh and dry weights, were quantified using a precision balance with a sensitivity of 0.001 g. Root length of each individual plant was measured using a precise ruler. Considering the homorhizous root system of barley, the total length of first‐order roots, comprising the primary root and all lateral roots emerging from it, was recorded. Measurements were taken carefully for each plant to ensure accuracy, and the total root length was documented in centimeters. Various root traits were calculated as follows: root area was determined using the formula “2 × √ (root volume × 3.14 × root length)” (Shaban et al. 2012), while root diameter was calculated as “√ [(4 × root fresh weight)/(root length × 3.14)]” (Hajabbasi 2001; Schenk and Barber 1979). Specific root length was derived by dividing root length by root dry weight (Huang et al. 1991; Mahanta et al. 2014), and root length density was calculated as the ratio of root length to soil volume (Mahanta et al. 2014; Mandal et al. 2003). Root specific mass was calculated by dividing root dry weight by soil volume (Hajabbasi 2001; Hasanabadi et al. 2010), and root tissue density was obtained by multiplying root dry weight by root volume (Paula and Pausas 2011). Root mass density was determined by dividing root fresh weight by soil volume (Hajabbasi 2001). Root fineness was calculated by dividing root length by root volume (Hajabbasi 2001), and root surface area density was determined as the product of root length, root diameter, and 3.14 (Ali et al. 2018). The root volume was determined by immersing the fresh plant roots in a graduated cylinder, and the change in water volume was measured.

### 2.4 Chlorophyll and Carotenoid

The chlorophyll and carotenoid content were measured using the method of Lichtenthaler and Wellburn (1983). A 25 mg sample of leaves was powdered in a Chinese mortar with liquid nitrogen, then homogenized in the dark with 2 mL of 96% ethanol. The solution was shaken and centrifuged for 10 min at 10,000 rpm at 4°C. After centrifugation, the supernatant was transferred to a plate and measured using an ELISA device (Bio Tek PowerWave) at wavelengths of 663, 646, and 470 nm. The concentrations of chlorophyll a, chlorophyll b, total chlorophyll, and carotenoids were calculated using the following formulas:

$$\mathrm{Chl}\;a=12.21\;\left(A_{663}\right)-2.81\;\left(A_{646}\right)$$

$$\mathrm{Chl}\;b=20.13\;\left(A_{646}\right)-5.1\;\left(A_{663}\right)$$

ChlT=Chla+Chlb

$$\mathrm{Car}=\left(1000\;A_{470}-3.27\;\left[\mathrm{Chl}\;a\right]-104\;\left[\mathrm{Chl}\;b\right]/227\right)$$

### 2.5 Statistical Analysis

To evaluate the differences among genotype groups and assess the effects of various variables, a one‐way analysis of variance (ANOVA) was performed for each of the traits under investigation. The analysis was conducted for the genotypic groups using SPSS software. Subsequently, the discriminant function for each genotypic group was performed step by step using XLSTAT. The value of the detected functions was calculated for each group. Additionally, all graphs were drawn using XLSTAT software. Factorial variance analysis was conducted using SPSS 22, with 5 replicates and nine control genotypes for root trait. Mean comparison was performed using the LSD method at the 5% and 1% levels. Correlation and cluster analysis (Euclidean distance) for traits were conducted using Ward's method with GraphPad Prism and ClustVis software (Metsalu and Vilo 2015).

## 3 Results

### 3.1 Root Grouping

**FIGURE 1:**
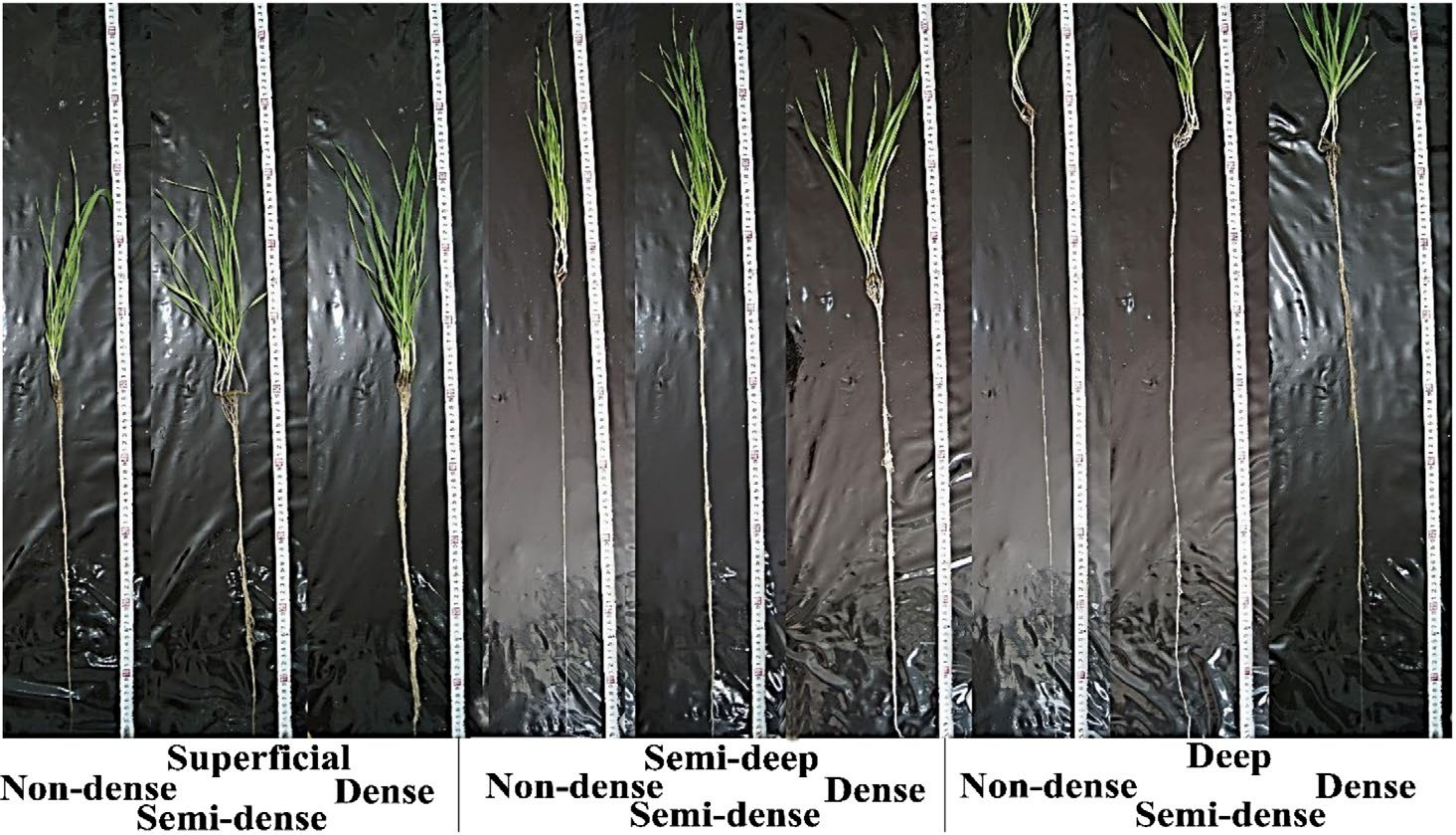
Classification of 114 wild barley genotypes into nine root groups under normal conditions based on root length and root tissue density using the 95% confidence interval. Root length categories: superficial, semi‐deep, deep; root tissue density categories: non‐dense, semi‐dense, dense.

**FIGURE 2:**
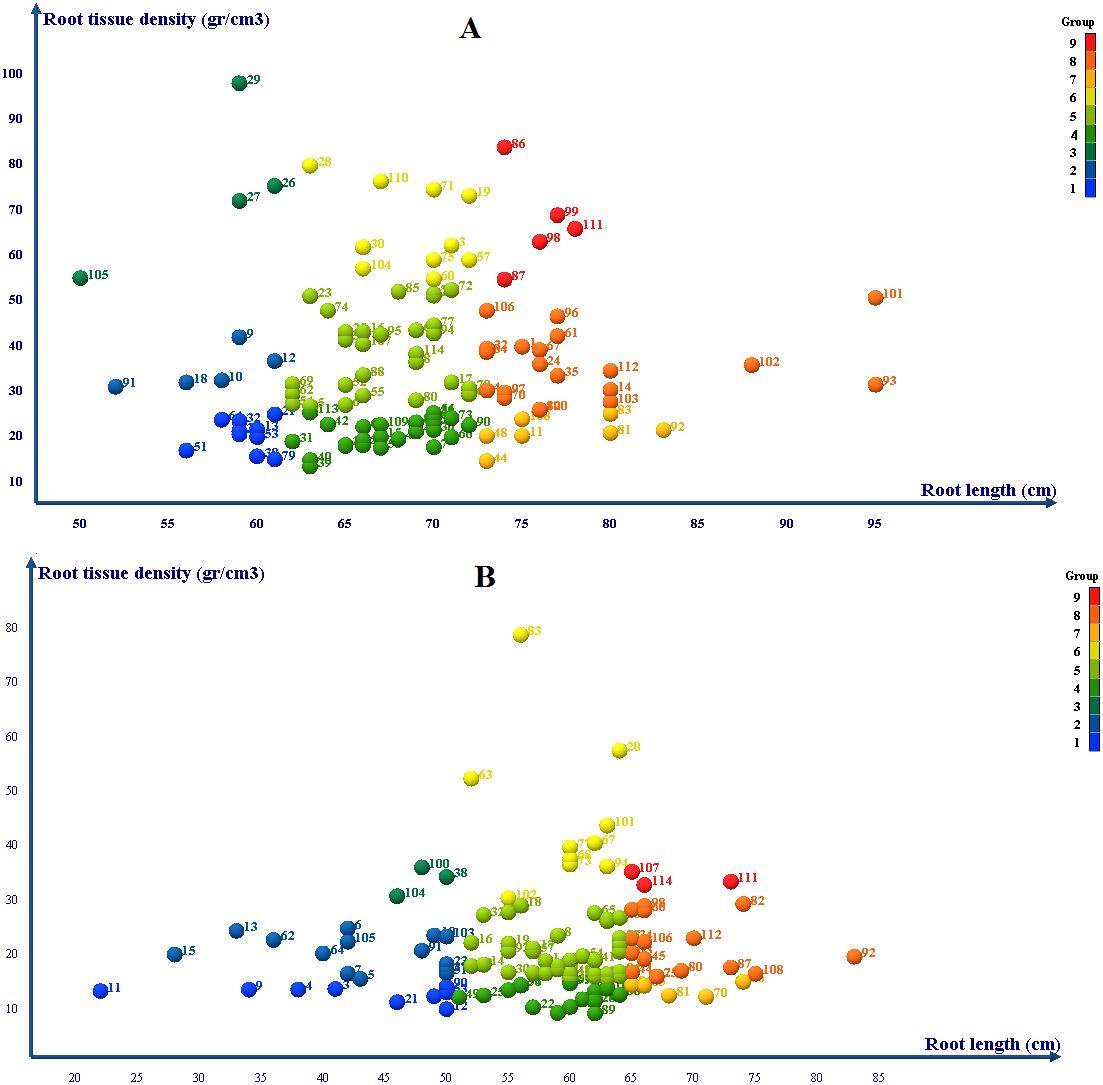
Grouping of genotypes investigated in roots based on the depth and density of root tissue. (A) normal condition, (B) water stress condition. 1: superficial non‐dense, 2: superficial semi‐dense, 3: superficial dense, 4: semi‐deep non‐dense, 5: semi‐deep semi‐dense, 6: semi‐deep dense, 7: deep non‐dense, 8: deep semi‐dense and 9: deep dense.

With this criterion, 114 genotypes were examined in terms of root length and root tissue density under water stress and normal conditions in nine groups including (superficial non‐dense, superficial semi‐dense, superficial dense, semi‐deep non‐dense, semi‐deep semi‐dense, semi‐deep dense, deep non‐dense, deep semi‐dense, and deep dense) (Table 1). The genotypes of the ninth group have the best root structure and architecture for better absorption of water from the surface and deep layers of the soil. Figure 1 shows the root groups under normal conditions. Figure 2 shows the grouping of roots based on the trait of root depth and root tissue density.

**TABLE 1:** Classification of 114 wild barley genotypes into nine root groups under normal and water stress conditions based on root length and root tissue density using the 95% confidence interval.

Genotype	Normal		Stress		Genotype	Normal		Stress	
	Root length	Root tissue density	Root length	Root tissue density		Root length	Root tissue density	Root length	Root tissue density
1	Deep	Semi‐dense	Semi‐deep	Semi‐dense	58	Semi‐deep	Non‐dense	Semi‐deep	Semi‐dense
2	Semi‐deep	Semi‐dense	Deep	Semi‐dense	59	Semi‐deep	Semi‐dense	Semi‐deep	Semi‐dense
3	Semi‐deep	Dense	Superficial	Non‐dense	60	Semi‐deep	Dense	Semi‐deep	Semi‐dense
4	Deep	Semi‐dense	Superficial	Non‐dense	61	Deep	Semi‐dense	Semi‐deep	Semi‐dense
5	Semi‐deep	Semi‐dense	Superficial	Semi‐dense	62	Semi‐deep	Semi‐dense	Superficial	Semi‐dense
6	Semi‐deep	Semi‐dense	Superficial	Semi‐dense	63	Semi‐deep	Semi‐dense	Semi‐deep	Dense
7	Semi‐deep	Non‐dense	Superficial	Semi‐dense	64	Superficial	Non‐dense	Superficial	Semi‐dense
8	Semi‐deep	Semi‐dense	Semi‐deep	Semi‐dense	65	Semi‐deep	Non‐dense	Semi‐deep	Semi‐dense
9	Superficial	Semi‐dense	Superficial	Non‐dense	66	Semi‐deep	Non‐dense	Deep	Semi‐dense
10	Superficial	Semi‐dense	Superficial	Semi‐dense	67	Deep	Semi‐dense	Semi‐deep	Dense
11	Deep	Non‐dense	Superficial	Non‐dense	68	Semi‐deep	Semi‐dense	Semi‐deep	Dense
12	Superficial	Semi‐dense	Superficial	Non‐dense	69	Semi‐deep	Semi‐dense	Semi‐deep	Non‐dense
13	Superficial	Non‐dense	Superficial	Semi‐dense	70	Deep	Semi‐dense	Deep	Non‐dense
14	Deep	Semi‐dense	Semi‐deep	Semi‐dense	71	Semi‐deep	Dense	Semi‐deep	Non‐dense
15	Semi‐deep	Non‐dense	Superficial	Semi‐dense	72	Semi‐deep	Semi‐dense	Semi‐deep	Non‐dense
16	Semi‐deep	Semi‐dense	Semi‐deep	Semi‐dense	73	Semi‐deep	Non‐dense	Semi‐deep	Dense
17	Semi‐deep	Semi‐dense	Semi‐deep	Semi‐dense	74	Semi‐deep	Semi‐dense	Semi‐deep	Non‐dense
18	Superficial	Semi‐dense	Semi‐deep	Semi‐dense	75	Semi‐deep	Dense	Deep	Semi‐dense
19	Semi‐deep	Dense	Semi‐deep	Semi‐dense	76	Semi‐deep	Semi‐dense	Semi‐deep	Semi‐dense
20	Semi‐deep	Non‐dense	Semi‐deep	Dense	77	Semi‐deep	Semi‐dense	Semi‐deep	Dense
21	Superficial	Non‐dense	Superficial	Non‐dense	78	Semi‐deep	Semi‐dense	Semi‐deep	Semi‐dense
22	Deep	Semi‐dense	Semi‐deep	Non‐dense	79	Superficial	Non‐dense	Semi‐deep	Semi‐dense
23	Semi‐deep	Semi‐dense	Superficial	Semi‐dense	80	Semi‐deep	Semi‐dense	Deep	Semi‐dense
24	Deep	Semi‐dense	Semi‐deep	Non‐dense	81	Deep	Non‐dense	Deep	Non‐dense
25	Semi‐deep	Semi‐dense	Semi‐deep	Non‐dense	82	Deep	Semi‐dense	Deep	Semi‐dense
26	Superficial	Dense	Semi‐deep	Non‐dense	83	Deep	Non‐dense	Semi‐deep	Dense
27	Superficial	Dense	Semi‐deep	Non‐dense	84	Deep	Semi‐dense	Semi‐deep	Semi‐dense
28	Semi‐deep	Dense	Semi‐deep	Non‐dense	85	Semi‐deep	Semi‐dense	Deep	Non‐dense
29	Superficial	Dense	Semi‐deep	Semi‐dense	86	Deep	Dense	Deep	Non‐dense
30	Semi‐deep	Dense	Semi‐deep	Semi‐dense	87	Deep	Dense	Deep	Semi‐dense
31	Semi‐deep	Non‐dense	Semi‐deep	Semi‐dense	88	Semi‐deep	Semi‐dense	Semi‐deep	Non‐dense
32	Superficial	Non‐dense	Semi‐deep	Semi‐dense	89	Semi‐deep	Non‐dense	Semi‐deep	Non‐dense
33	Semi‐deep	Non‐dense	Deep	Semi‐dense	90	Semi‐deep	Non‐dense	Superficial	Non‐dense
34	Semi‐deep	Non‐dense	Deep	Semi‐dense	91	Superficial	Semi‐dense	Superficial	Semi‐dense
35	Deep	Semi‐dense	Semi‐deep	Semi‐dense	92	Deep	Non‐dense	Deep	Semi‐dense
36	Semi‐deep	Non‐dense	Semi‐deep	Semi‐dense	93	Deep	Semi‐dense	Semi‐deep	Semi‐dense
37	Superficial	Non‐dense	Superficial	Semi‐dense	94	Semi‐deep	Semi‐dense	Semi‐deep	Dense
38	Superficial	Non‐dense	Superficial	Dense	95	Semi‐deep	Semi‐dense	Deep	Semi‐dense
39	Semi‐deep	Non‐dense	Semi‐deep	Semi‐dense	96	Deep	Semi‐dense	Deep	Non‐dense
40	Semi‐deep	Non‐dense	Semi‐deep	Semi‐dense	97	Deep	Semi‐dense	Semi‐deep	Semi‐dense
41	Semi‐deep	Non‐dense	Semi‐deep	Semi‐dense	98	Deep	Dense	Deep	Semi‐dense
42	Semi‐deep	Non‐dense	Semi‐deep	Non‐dense	99	Deep	Dense	Semi‐deep	Non‐dense
43	Semi‐deep	Non‐dense	Semi‐deep	Semi‐dense	100	Deep	Semi‐dense	Superficial	Dense
44	Deep	Non‐dense	Deep	Semi‐dense	101	Deep	Semi‐dense	Semi‐deep	Dense
45	Semi‐deep	Non‐dense	Deep	Semi‐dense	102	Deep	Semi‐dense	Semi‐deep	Dense
46	Semi‐deep	Non‐dense	Semi‐deep	Semi‐dense	103	Deep	Semi‐dense	Superficial	Semi‐dense
47	Semi‐deep	Non‐dense	Semi‐deep	Semi‐dense	104	Semi‐deep	Dense	Superficial	Dense
48	Deep	Non‐dense	Semi‐deep	Semi‐dense	105	Superficial	Dense	Superficial	Semi‐dense
49	Superficial	Non‐dense	Semi‐deep	Non‐dense	106	Deep	Semi‐dense	Deep	Semi‐dense
50	Semi‐deep	Non‐dense	Superficial	Non‐dense	107	Semi‐deep	Semi‐dense	Deep	Dense
51	Superficial	Non‐dense	Superficial	Semi‐dense	108	Deep	Non‐dense	Deep	Semi‐dense
52	Semi‐deep	Semi‐dense	Semi‐deep	Semi‐dense	109	Semi‐deep	Non‐dense	Semi‐deep	Non‐dense
53	Superficial	Non‐dense	Superficial	Non‐dense	110	Semi‐deep	Dense	Semi‐deep	Semi‐dense
54	Semi‐deep	Semi‐dense	Semi‐deep	Semi‐dense	111	Deep	Dense	Deep	Dense
55	Semi‐deep	Semi‐dense	Semi‐deep	Semi‐dense	112	Deep	Semi‐dense	Deep	Semi‐dense
56	Semi‐deep	Non‐dense	Semi‐deep	Non‐dense	113	Semi‐deep	Non‐dense	Semi‐deep	Semi‐dense
57	Semi‐deep	Dense	Semi‐deep	Semi‐dense	114	Semi‐deep	Semi‐dense	Deep	Dense

*Note:* Root length categories: superficial, semi‐deep, deep; root tissue density categories: non‐dense, semi‐dense, dense.

The results showed that in normal conditions, 19 genotypes had superficial roots, 63 genotypes had semi‐deep roots, and 32 genotypes had deep roots. Also, 42 genotypes had non‐dense, 53 genotypes had semi‐dense, and 19 genotypes had dense structure. That genotypes 86, 87, 98, 99, and 111 were placed in the ninth group, deep and dense structure.

In the water stress condition, the structure of the genotypes changed. In such a way that the structure of many genotypes was different from normal conditions. Under water stress conditions, 26 genotypes had superficial roots, 64 genotypes had semi‐deep roots, and 24 genotypes had deep roots. Also, 30 genotypes had non‐dense roots, 67 semi‐dense genotypes, and 16 dense root genotypes were found. The genotypes 107, 111, and 114 were placed in the ninth group, deep and dense structure (Table 1).

**FIGURE 3:**
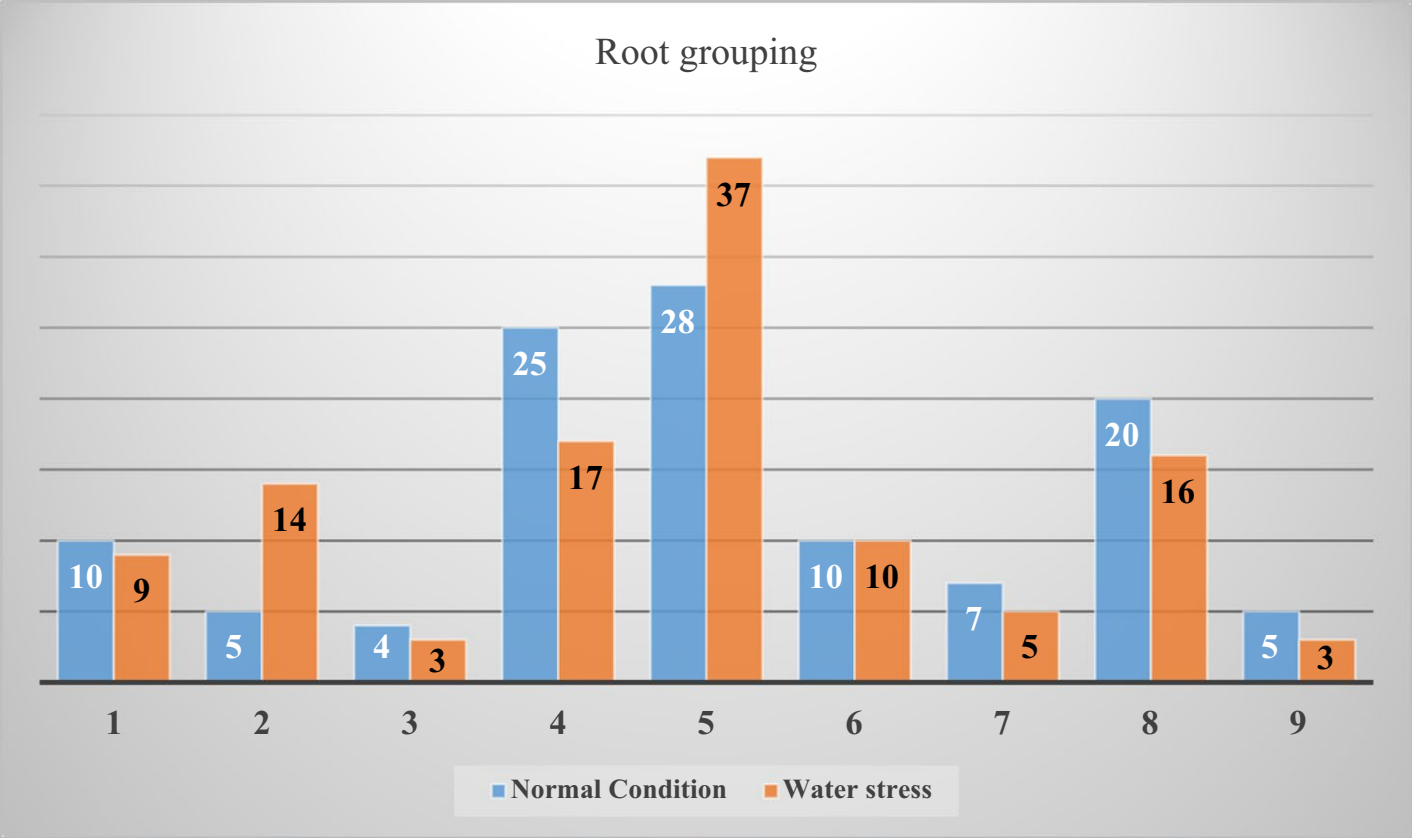
Number of wild barley genotypes in nine root groups under normal and water stress conditions. 1: superficial non‐dense, 2: superficial semi‐dense, 3: superficial dense, 4: semi‐deep non‐dense, 5: semi‐deep semi‐dense, 6: semi‐deep dense, 7: deep non‐dense, 8: deep semi‐dense and 9: deep dense.

The results showed that in normal condition, 10 genotypes had superficial non‐dense, 5 genotypes had superficial semi‐dense, 4 genotypes had superficial dense, 25 genotypes had semi‐deep non‐dense, 28 genotypes had semi‐deep semi‐dense, 10 genotypes had semi‐deep dense, 7 genotypes had deep non‐dense, 20 genotypes had deep semi‐dense, and 5 genotypes had deep dense (Figure 3).

Also under water stress conditions, 9 genotypes had superficial non‐dense, 14 genotypes had superficial semi‐dense, 3 genotypes had superficial dense, 17 genotypes had semi‐deep non‐dense, 37 genotypes had semi‐deep semi‐dense, 10 genotypes had semi‐deep dense, 5 genotypes had deep non‐dense, 16 genotypes had deep semi‐dense, and 3 genotypes had deep dense (Figure 3). Genotype 111 had the same structure (deep and dense) in both water stress and normal conditions.

The results of the analysis of variance for nine genotypic groups created under normal conditions showed a significant difference between the different groups in terms of traits such as root length, root volume, root dry weight, root diameter, specific root length, root length density, root specific mass, root tissue density, root surface area density, and root area (Table 2). In addition, water stress variance analysis for 9 genotypic groups showed significant differences between groups in terms of all traits (Table 3).

**TABLE 2:** Results of variance analysis of root groups for wild barley under normal conditions.

S.O.V	Mean square													
	Df	Root length	Root fresh weight	Root volume	Root dry weight	Root fineness	Root diameter	Specific root length	Root length density	Root specific mass	Root tissue density	Root mass density	Root surface area density	Root area
Groups	8	600.859**	0.216^ns^	0.000011**	0.024**	830.182^ns^	0.0000236**	22,4909.073**	0.000211**	0.0000000083**	3737.892**	0.00000008^ns^	1.186**	0.272**
Error	105	15.772	0.125	0.00000002	0.004	1185.531	0.0000053	62,457.830	0.0000005	0.0000000013	54.700	0.00000004	0.238	0.035

*Note:* Root groups were classified based on root length and root tissue density using the 95% confidence interval. * and ** are respectively significant at the 5% and 1% probability level and ^ns^ are not significant.

**TABLE 3:** Results of variance analysis of root groups for wild barley under water stress.

S.O.V	Mean square													
	Df	Root length	Root fresh weight	Root volume	Root dry weight	Root fineness	Root diameter	Specific root length	Root length density	Root specific mass	Root tissue density	Root mass density	Root surface area density	Root area
Groups	8	1067.340**	0.097*	0.000009**	0.001*	14,524.729*	0.0000251**	28,2144.653*	0.000375**	0.0000000004*	1132.663**	0.00000003*	0.149*	0.858**
Error	105	26.273	0.045	0.000001	0.001	5694.743	0.0000043	11,1383.150	0.000009	0.0000000002	28.760	0.00000002	0.071	0.186

*Note:* Root groups were classified based on root length and root tissue density using the 95% confidence interval. * and ** are respectively significant at the 5% and 1% probability level and ^ns^ are not significant.

**FIGURE 4:**
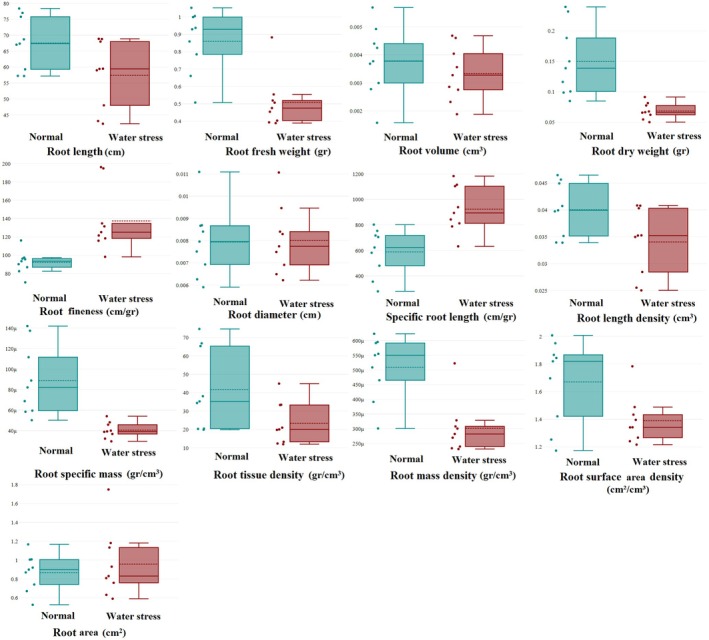
Mean root traits of wild barley genotypes in nine root groups under normal and water stress conditions. Root groups were established based on root length and root tissue density using the 95% confidence interval.

The results showed that, in normal conditions, the mean of all root traits, except for root fineness, root area and specific root length, is higher than in water stress conditions (Figure 4).

In normal conditions, Group 2 (superficial semi‐dense) had the highest root fineness and specific root length. Group 3 (superficial dense) had the highest root diameter and root tissue density, while Group 7 (deep non‐dense) had the highest root volume and root area. Group 8 (deep semi‐dense) had the highest root length and root length density. Lastly, Group 9 (deep dense) had the highest root fresh weight, root dry weight, root specific mass, root mass density, and root surface area density.

Group 1 (superficial non‐dense) had the lowest root dry weight, root specific mass, root tissue density, and root surface area density. Group 2 (superficial semi‐dense) had the lowest root length, root fresh weight, root volume, root length density, root mass density, and root area. Group 3 (superficial dense) had the lowest root fineness and specific root length. Additionally, Group 4 (semi‐deep non‐dense) had the lowest root diameter (Figure 4).

In water stress conditions, Group 1 (superficial non‐dense) had the highest root diameter, while Group 6 (semi‐deep dense) had the highest root fineness and root tissue density. Group 7 (deep non‐dense) had the highest root volume and root area. Group 8 (deep semi‐dense) had the highest root length, specific root length, and root length density. Lastly, Group 9 (deep dense) had the highest root fresh weight, root dry weight, root specific mass, root mass density, and root surface area density.

Group 1 (superficial non‐dense) had the lowest root length, root dry weight, root fineness, root length density, root specific mass, and root tissue density. Group 2 (superficial semi‐dense) had the lowest root fresh weight and root mass density. Group 3 (superficial dense) had the lowest specific root length and root area. Additionally, Group 6 (semi‐deep dense) had the lowest root volume, root diameter, and root surface area density (Figure 4).

The purpose of the discriminant function is to investigate how to separate two or more groups of genotypes based on the attributes evaluated on several variables. The discriminant function was performed step by step to separate nine genotypic groups created under both water stress and normal conditions. Based on the obtained results, under normal and water stress conditions, respectively, the three main discriminant functions explained 95.3% and 94.2% of the total variance. Therefore, new individuals can be assigned to these groups (Table 4).

**TABLE 4:** Specific values and the proportion of variance explained by the first two discriminant functions for the nine root groups of wild barley genotypes.

	Eigenvalues				
	Function	Eigenvalue	% of Variance	Cumulative %	Canonical correlation
Water stress	5.599^a^	55.8	55.8	0.921	5.599^a^
	2.899^a^	28.9	84.7	0.862	2.899^a^
	0.946^a^	9.4	94.2	0.697	0.946^a^
Normal	7.173^a^	63.3	63.3	0.937	7.173^a^
	3.026^a^	26.7	90.0	0.867	3.026^a^
	0.599^a^	5.3	95.3	0.612	0.599^a^

	Wilks' lambda				
	Test of function(s)	Wilks' lambda	Chi‐square	Df	Sig.
Water stress	1 through 8	0.012	458.141	88	0.000
	2 through 8	0.077	263.790	70	0.000
	3 through 8	0.301	123.645	54	0.000
Normal	1 through 8	0.012	460.541	80	0.000
	2 through 8	0.095	243.107	63	0.000
	3 through 8	0.384	98.952	48	0.000

*Note:* The discriminant functions were calculated based on root length and root tissue density traits under normal and water stress conditions. The superscript “a” in the Eigenvalue and Canonical correlation columns corresponds to SPSS‐generated notations for the extracted discriminant functions and has no statistical or interpretive meaning beyond this.

The standardized coefficients of traits in the first and second discriminant functions are listed in Table 5. In water stress conditions, based on the values of detection functions extracted for each group, the first function can be referred to as root length density and root length, while the second function can be referred to as root tissue density. Also, in normal conditions, the first function is referred to as root tissue density and the second function is referred to as root length and root tissue density. These attributes have the highest coefficients in determining the functions. The values of focal functions extracted for various genotypic groups under both water stress and normal conditions can be observed in Table 6.

**TABLE 5:** Standardized coefficients of key root traits in the first and second discriminant functions for separating the nine root groups of wild barley genotypes.

	Structure matrix					
	Function					
	Water stress			Normal		
	1	2	3	1	2	3
Root length density	0.604*	0.590	0.136	−0.098	0.966*	−0.106
Root length	0.604*	0.590	0.136	−0.098	0.966*	−0.106
Root tissue density	0.478	−0.739*	0.378	0.836*	0.234	0.031
Root fineness	0.155	−0.016	0.095	−0.028	−0.024	−0.159
Root dry weight	0.133	−0.137	−0.068	0.228	0.176	0.107
Root specific mass	0.133	−0.137	−0.068	0.228	0.176	0.107
Specific root length	0.044	0.229	0.080	−0.174	−0.079	−0.113
Root volume	−0.152	0.386*	0.061	−0.165	0.100	0.388*
Root area	0.063	0.298	0.180	−0.204	0.252	0.383*
Root mass density	0.057	0.107	−0.166	0.053	0.168	0.138
Root fresh weight	0.057	0.107	−0.166	0.053	0.168	0.138
Root diameter	−0.234	−0.121	−0.171	0.186	0.015	−0.202
Root surface area density	0.033	0.135	−0.140	0.154	0.205	−0.230

*Note:* The discriminant functions were based on root length and root tissue density measured under normal and water stress conditions. The asterisk (*) indicates the root traits with the highest standardized coefficients in each discriminant function, representing the most influential traits for group separation.

**TABLE 6:** Centroid values of the first and second discriminant functions for the nine root groups of wild barley genotypes.

Functions at group centroids						
Groups	Function					
	Water stress			Normal		
	1	2	3	1	2	3
1	−4.025	−0.277	0.115	−2.079	−2.702	0.360
2	−2.732	−2.031	−0.824	0.981	−3.139	−2.138
3	−0.269	−3.393	0.050	7.477	−1.946	1.462
4	−1.666	1.625	0.932	−2.340	−0.514	0.702
5	0.335	0.292	−0.384	0.474	−0.462	−0.518
6	3.187	−2.987	1.944	4.357	0.365	0.177
7	0.595	2.810	0.922	−2.844	1.884	0.431
8	2.575	1.378	−0.422	−0.645	2.408	−0.528
9	5.053	−1.183	−2.864	4.087	2.260	0.794

*Note:* These values were calculated based on root length and root tissue density traits under normal and water stress conditions.

**FIGURE 5:**
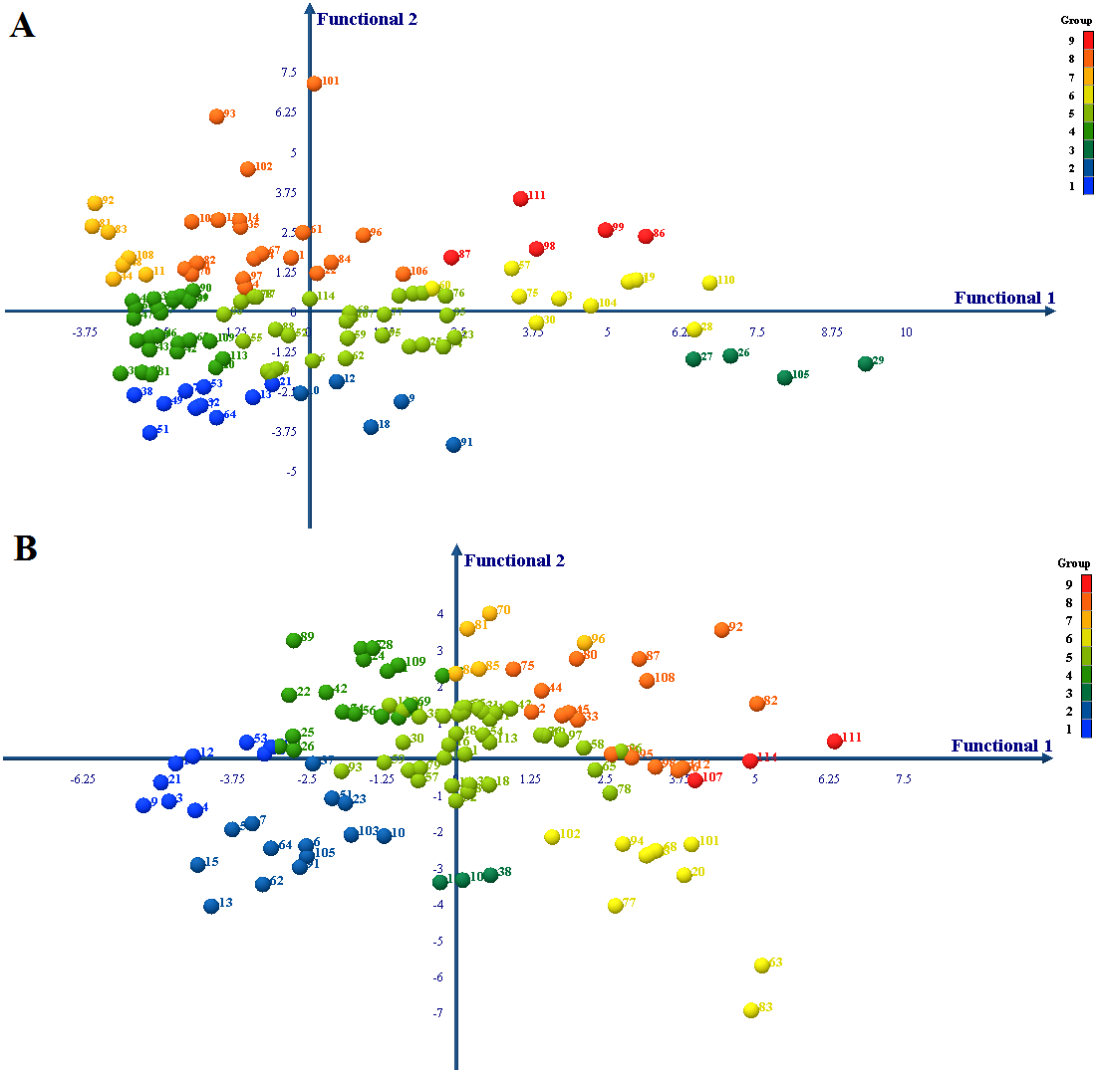
Bi‐plot diagram showing the distribution of 114 wild barley genotypes based on discriminant analysis of root traits under normal (A) and water stress (B) conditions. The discriminant analysis was performed using root length and root tissue density, and genotypes were classified into nine root groups. 1: Superficial non‐dense, 2: Superficial semi‐dense, 3: Superficial dense, 4: Semi‐deep non‐dense, 5: Semi‐deep semi‐dense, 6: Semi‐deep dense, 7: Deep non‐dense, 8: Deep semi‐dense and 9: Deep dense.

Considering the significant contribution of the first and second functions in determining the variance between groups, a bi‐plot diagram of 9 groups was created based on these two functions (Figure 5). This bi‐plot allows us to observe the relative positions of the groups. As depicted in the figure, the different groups are largely distinct, thereby confirming the grouping determined by the crucial trait of root depth and density.

### 3.2 Correlation

**FIGURE 6:**
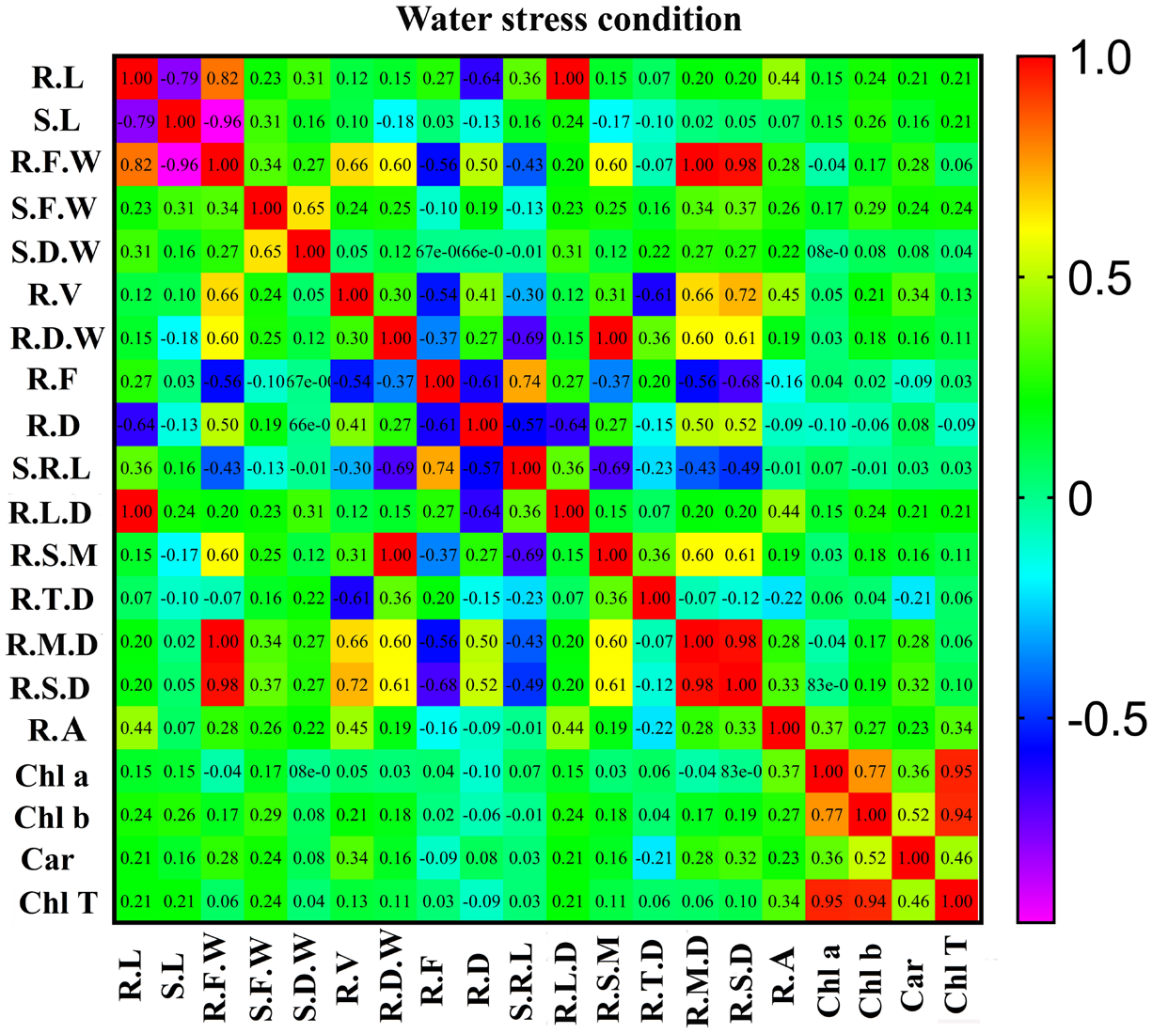
Correlation matrix showing relationships between root and seedling traits of the nine root groups of wild barley under water stress conditions. Root groups were established based on the classification of 114 genotypes using root length and root tissue density. Car, carotenoid; Chl a, chlorophyll a; Chl b, chlorophyll b; Chl T, total chlorophyll; R.A, root area; R.D, root diameter; R.D.W, root dry weight; R.F, root fineness; R.F.W, Root fresh weight; R.L, root length; R.L.D, root length density; R.M.D., root mass density, R.S.D., root surface area density; R.S.M, root specific mass, R.T.D, root tissue density; R.V, root volume; S.D.W, root dry weight; S.F.W, Root fresh weight; S.L, seedling length; S.R.L, specific root length.

In water stress conditions (Figure 6), the correlation between root length and seedling dry weight, root fineness, root diameter, specific root length, root length density, root area, and chlorophyll b was positive and significant at the 1% level, as well as with seedling traits. Seedling length, root fresh weight, seedling fresh weight, root mass density, root surface area density, carotenoid, and total chlorophyll were positively correlated and significant at a 5% level. The correlation of seedling length with seedling fresh weight, root length density, chlorophyll b, and total chlorophyll traits was positive and significant. The correlation of root fresh weight with seedling fresh weight, seedling dry weight, root volume, root dry weight, root length density, root specific mass, root mass density, root surface area density, root area, and carotenoid traits was positive and significant, and the correlation with root fineness and specific root length traits was observed to be negative and significant. The correlation of seedling fresh weight with seedling dry weight, root volume, root dry weight, root specific mass, root mass density, root surface area density, root area, chlorophyll b, carotenoid, and total chlorophyll was positive and significant. The correlation of seedling dry weight with root length density, root tissue density, root length density, root surface area density, and root area was positive and significant. The correlation of root dry weight with root specific mass, root tissue density, root mass density, root surface area density, and root area was positive and significant, and with root fineness and specific root length was negative and significant.

**FIGURE 7:**
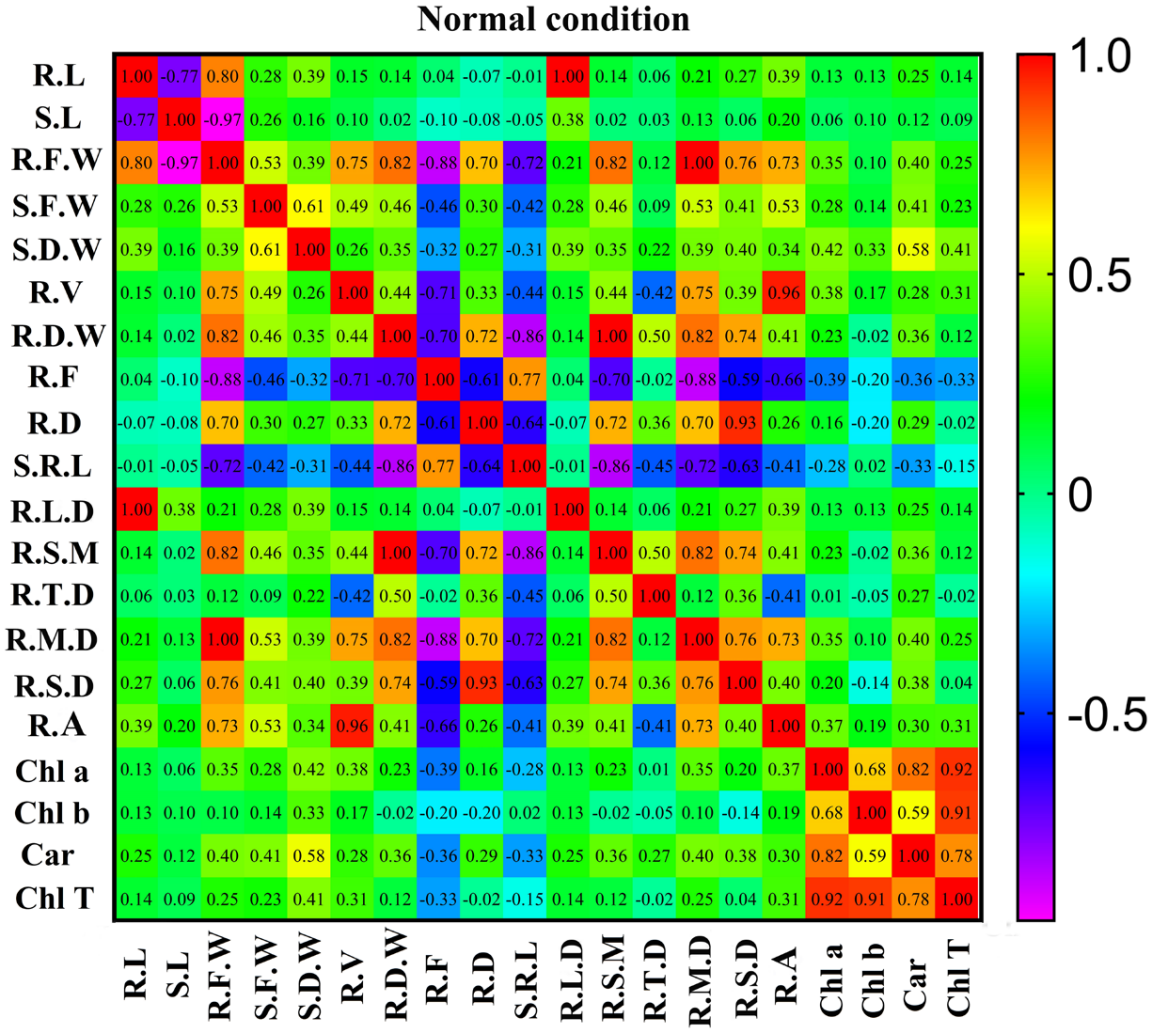
Correlation matrix showing relationships between root and seedling traits of the nine root groups of wild barley under normal conditions. Root groups were established based on the classification of 114 genotypes using root length and root tissue density. Car, carotenoid; Chl a, chlorophyll a; Chl b, chlorophyll b; Chl T, total chlorophyll; R.A, root area; R.D, root diameter; R.D.W, root dry weight; R.F, root fineness; R.F.W, Root fresh weight; R.L, root length; R.L.D, root length density; R.M.D., root mass density, R.S.D., root surface area density; R.S.M, root specific mass, R.T.D, root tissue density; R.V, root volume; S.D.W, root dry weight; S.F.W, Root fresh weight; S.L, seedling length; S.R.L, specific root length.

In normal conditions (Figure 7), the correlation between root length and seedling length, root fresh weight, seedling fresh weight, root length density, root mass density, root surface area density, root area, and carotenoid traits is positive and at a significant level. The correlation of seedling length with seedling fresh weight, density, root length, and root area was positive and significant. The correlation of root fresh weight with the traits of seedling fresh weight, seedling dry weight, root volume, and root dry weight, root density, root length, and root area was positive and significant, and the correlation with the traits of root fineness and specific root length was negative and significant. Correlation of seedling fresh weight with traits of plant dry weight, root volume, root dry weight, root diameter, root mass density, root surface area density, root area, root density, root length, root specific mass, chlorophyll a, carotenoid, and total chlorophyll was positive and significant, and with root fineness and specific root length, this correlation was negative and significant. The correlation of seedling dry weight with root volume, root dry weight, root length density, root specific mass, root tissue density, root mass density, root surface area density, root area, chlorophyll a, chlorophyll b, carotenoid, and total chlorophyll is positive and significant, and with root fineness and specific root length, this correlation was negative and significant. The correlation of root dry weight with root specific mass, root tissue density, root mass density, root surface area density, root area, chlorophyll a, and carotenoid was positive and significant, and with root fineness and specific root length, this correlation was negative and significant.

### 3.3 Cluster Analysis

**FIGURE 8:**
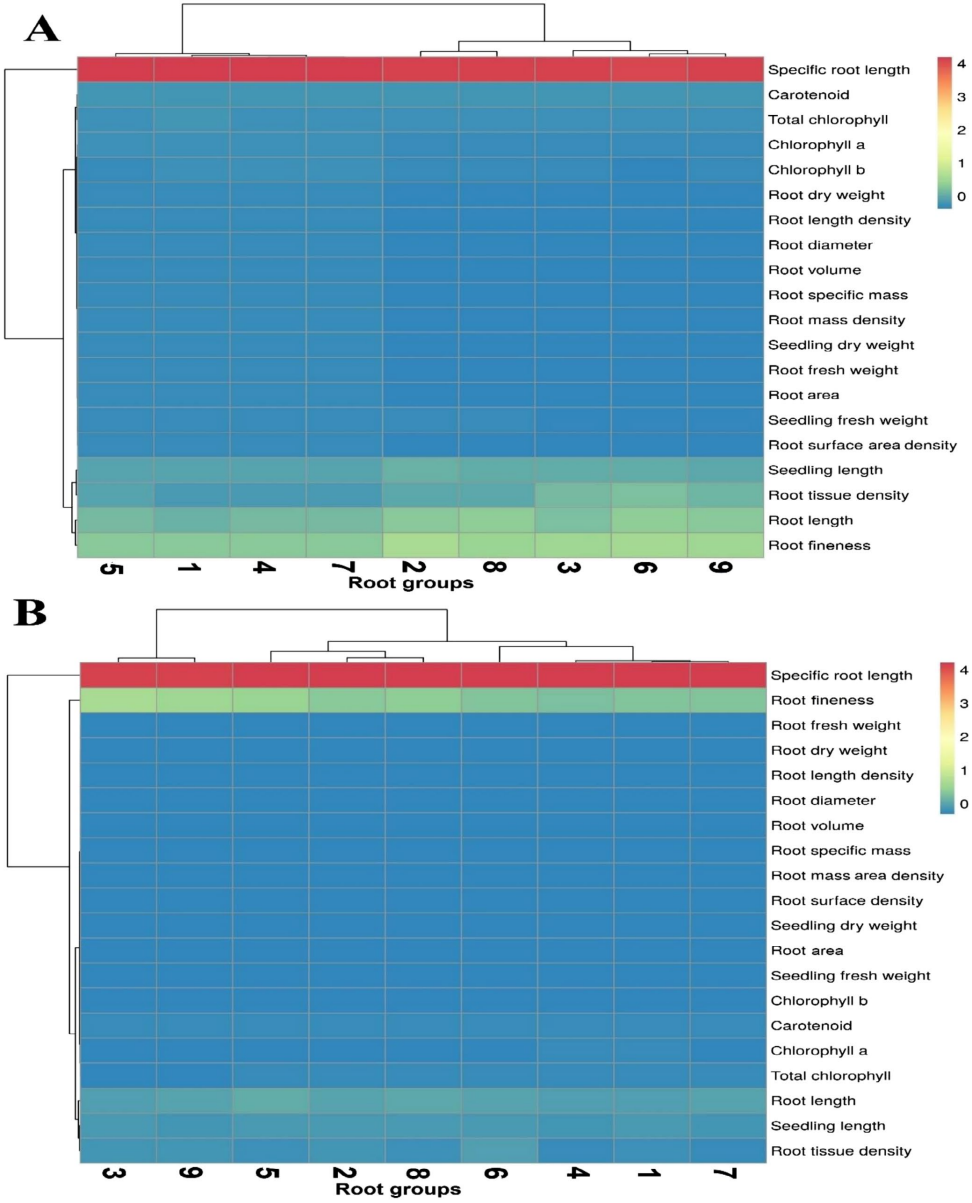
Dendrogram and heatmap showing cluster analysis of wild barley root groups under normal (A) and water stress (B) conditions. Clustering was performed using Ward's method based on root and seedling traits. In the heatmap, traits are organized along the horizontal axis, and the root groups are shown along the vertical axis. The color gradient represents the relative magnitude of each trait across the groups. 1: Superficial non‐dense, 2: Superficial semi‐dense, 3: Superficial dense, 4: Semi‐deep non‐dense, 5: Semi‐deep semi‐dense, 6: Semi‐deep dense, 7: Deep non‐dense, 8: Deep semi‐dense and 9: Deep dense.

To group the studied nine groups, cluster analysis by the WARD method was used. Based on cluster analysis, the nine groups were placed in three clusters in water stress and normal conditions respectively (Figure 8).

## 4 Discussion

The considerable diversity observed among the 114 wild barley ( *H. spontaneum* ) genotypes, particularly in root traits, provides valuable insights for potential applications in breeding programs aimed at improving drought tolerance. This variability not only enhances our understanding of the inherent capabilities of these genotypes but also facilitates subsequent molecular studies and cultivar evaluations. An optimal root system is influenced by both soil and climatic factors; however, this study focused on root characteristics during the peak vegetative growth phase (approximately 4–6 weeks), under controlled water deficit conditions, without including environmental parameters such as soil pH, nutrient availability, or long‐term plant performance. Despite these limitations, the documented variation establishes a foundational resource for field evaluations and breeding efforts, supporting the identification of drought tolerance genotypes that may contribute to improved yield stability under water stress conditions. Future research integrating long‐term growth, yield assessment, and environmental interactions will be essential to translate these root trait differences into practical agricultural benefits and to validate their utility in breeding programs. Moreover, the high level of diversity observed provides a natural buffer against adverse environmental conditions, highlighting the adaptive potential of wild barley germplasm. The findings of Comas et al. (2013) further substantiate this notion, suggesting that early‐stage evaluation of root traits can serve as reliable indicators for predicting seed yield by the conclusion of the growing season. Furthermore, transgenic barley, engineered for root‐specific expression of a cytokinin degrading gene, demonstrated an expanded root system, which in turn enhanced drought tolerance and nutrient uptake without negatively affecting shoot growth or seed yield (Ramireddy et al. 2018).

Root system architecture (RSA) is a dynamic trait that is significantly influenced by environmental factors, including soil moisture, temperature, nutrient availability, and pH. The various characteristics of the root system enable plants to thrive under diverse environmental conditions and adapt to these conditions throughout the growth period. As the global population continues to rise, there is an increasing demand for enhanced production, which can be achieved through the development of high‐yielding cultivars. This is traditionally accomplished using common breeding approaches focused on improving grain and fodder yields. These breeding strategies typically select superior individuals based on desirable growth traits such as leaf area, seed number, and resistance to pests and diseases. However, breeding strategies targeting root traits emphasize the identification of structural characteristics that allow plants to more effectively access water and nutrients. By prioritizing root traits in breeding programs, it is possible to close the yield gap caused by suboptimal root systems, particularly in the face of climate change (Mann et al. 2023). Notably, significant variations in RSA traits were observed among 300 barley accessions, especially in root length and number, which are critical for resistance to soil acidity (Abebe et al. 2023).

In this study, 114 genotypes of wild barley were evaluated for their root architecture under both normal and water stress conditions. The genotypes were classified into nine distinct root structure categories based on root depth and tissue density, ranging from surface non‐dense roots to deep dense roots. Under normal conditions, 19 genotypes exhibited surface roots, 63 had semi‐deep roots, and 32 had deep roots. Additionally, 42 genotypes displayed non‐dense root structures, 53 had semi‐dense roots, and 19 had dense root systems. Notably, genotypes 86, 87, 98, 99, and 111 demonstrated the best root structures, characterized as deep and dense, which are considered optimal for water absorption. Under water stress conditions, these genotypes (107, 111, 114) retained their position in the deep and dense group, although the root structure of many genotypes changed compared to the normal conditions. The categorization of genotypes based on root tissue density and root length provided a comprehensive framework to examine how water stress affects various root traits. Barley roots exhibit adaptive responses to water stress, including alterations in architecture, anatomy, and biophysical properties. Under water deficit conditions, significant variations in nodal root traits were observed, with a reduction in cross‐sectional areas of main‐shoot roots and an increase in tiller root areas (Oyiga et al. 2020).

Significant differences in root traits, including root length, volume, diameter, dry weight, and surface area, were observed among the different root groups under both normal and water stress conditions. Under water stress, most root traits (with the exceptions of root fresh weight, root area, and specific root length) exhibited a decrease. This suggests that water stress negatively affects root traits involved in water storage and uptake. The reduction in these traits can be attributed to the plant's diminished capacity to absorb and retain water during drought conditions, which consequently impacts root growth and development. Group 9, characterized by deep and dense roots, demonstrated the highest values for root fresh weight, dry weight, specific root mass, and surface area density, both under normal and water stress conditions.

Conversely, genotypes with surface non‐dense roots exhibited the lowest values in most root traits. Understanding root structure, which contributes to higher yield and improved stress tolerance, is a vital tool for plant breeders to select ideal individuals for breeding programs (Pace et al. 2014). A study comparing the early stage shoot and root growth of barley landraces and modern cultivars under drought conditions found that drought‐induced reductions were more pronounced in shoot growth than in root growth. Some genotypes redirected growth towards lateral roots under dry soil conditions, reflecting genetic diversity in early growth dynamics (Boudiar et al. 2020). Ma et al. (2010) emphasized that the type of root system and its response to drought stress are crucial factors for drought tolerance. A larger root system is more advantageous than a smaller one for water storage, and strategies for improving drought tolerance focus on selecting generations with larger root systems.

In this study, the results showed a significant decrease in chlorophyll and carotenoid levels under water stress conditions. This reduction was more pronounced in drought‐sensitive genotypes compared to drought‐tolerant ones. Notably, drought‐tolerant genotypes exhibited a less significant decrease in chlorophyll and carotenoid levels, suggesting that these genotypes possess a greater ability to maintain photosynthetic function even under water stress. Water stress in barley affects both root structure and chlorophyll content. Under water stress, barley roots undergo structural changes, such as increased suberization, which reduces hydraulic conductivity and aids in water retention (Kreszies et al. 2019). Moreover, water stress causes a decrease in total chlorophyll content, which is more pronounced under severe stress conditions (Salekjalali et al. 2012). This reduction in chlorophyll content is linked to impaired photosynthetic efficiency and reduced plant growth (Alghabari and Ihsan 2018).

Based on the results of the discriminant function analysis, the first function revealed a greater disparity in the values obtained for the established groups. This finding provides a solid foundation for future research aimed at selecting, improving, and cultivating wild barley genotypes from Iran to enhance their resilience to water stress and adaptability to climate change. The first and second discriminant functions accounted for over 94% of the variance among the genotypic groups under both normal and stress conditions. This analysis emphasized the significance of root tissue density, root length, and root length density as key traits for differentiating between groups. In a similar context, discriminant function analysis (DFA) effectively distinguished Syrian from Japanese barley cultivars based on morphological traits, including root length and shoot dry weight (Kanbar 2011).

The correlation analysis revealed strong associations between various root and seedling traits. Under both normal and water stress conditions, positive correlations were observed between root length and seedling fresh weight, root length density, and root surface area density. These traits also exhibited positive correlations with chlorophyll content and carotenoid levels, suggesting that efficient root architecture enhances water absorption and overall plant health under stress conditions. Additionally, traits such as root diameter, specific root length, and root fineness showed negative correlations with several other root traits, indicating trade‐offs between different aspects of root morphology and physiology. The results from trait correlation and cluster analysis, conducted under both water stress and normal conditions, highlighted the varying reactions of the genotypic groups to water stress. Studies on cultivated and wild barley genotypes have shown that while a vigorous root system is not always directly linked to higher grain yield, it plays a crucial role in ensuring yield stability under water stress (Barati et al. 2020).

This study underscores the substantial genetic variability in root architecture among wild barley genotypes, with certain root traits proving particularly advantageous for water acquisition under both optimal and water stress conditions. These findings offer valuable guidance for breeding programs aimed at enhancing drought tolerance in barley, positioning root architecture as a key selection criterion. Nevertheless, caution is warranted when extrapolating these results to other cereal species, as species‐specific differences in root development and environmental responses may limit direct applicability. Despite this, the observed variation in barley roots provides a useful reference for future comparative studies targeting drought tolerance root traits in related cereals. The differences in root characteristics identified here may offer preliminary insights for selecting genotypes with improved water uptake and drought tolerance, potentially contributing to crop performance under water stress conditions projected under future climate scenarios. Furthermore, the identification of genetic markers associated with drought tolerance root traits could facilitate marker‐assisted selection, accelerating the development of cultivars with enhanced drought tolerance. Integrating detailed root phenotyping with molecular analyses in future studies will be essential to fully realize the potential of these traits for practical agricultural applications.

## 5 Conclusion

The present study aimed to evaluate root traits of 114 wild barley genotypes under both normal and water stress conditions, considering traits such as root length, root tissue density, and root architecture. The analysis demonstrated that root structure varies significantly under water stress, leading to changes in genotype categorization compared to normal conditions. Notably, the deep and dense root structure (Group 9) showed the highest potential for water absorption, consistently performing well across both conditions. The discriminant analysis revealed that the root traits, particularly root tissue density and root length, were key factors in differentiating genotypes under both normal and stress conditions, explaining 95.3% and 94.2% of the total variance, respectively. These findings emphasize the importance of root architecture as a determinant of drought tolerance and provide a framework for selecting genotypes with enhanced water absorption capabilities. The grouping of genotypes using the WARD method confirmed the distinctiveness of the root traits under both conditions, with clustering showing clear separation between groups based on root depth and density. Moreover, the correlation analysis underscored the interdependence of root traits with seedling growth and chlorophyll content, which further supports the relationship between root morphology and drought tolerance. The genotypes within Group 9, in particular, were identified as potential candidates for further breeding programs aimed at improving drought tolerance. This comprehensive analysis of root traits not only addresses the main question posed in the study but also contributes valuable insights into the complex interplay between root structure and water stress tolerance in wild barley genotypes.

## Funding

The authors have nothing to report.

## Conflicts of Interest

The authors declare no conflicts of interest.

## Supporting information


**Table S1:** List of wild barley genotypes used in this study, along with their collection sites and precise geographic coordinates (latitude and longitude).

## Data Availability

The data that support the findings of this study are openly available in Zenodo at https://doi.org/10.5281/zenodo.15034069, reference number 15034069.
